# Angiotensin-Converting Enzyme-2 (*ACE-2*) with Interferon-Induced Transmembrane Protein-3 (*IFITM-3*) Genetic Variants and Interleukin-6 as Severity and Risk Predictors among COVID-19 Egyptian Population

**DOI:** 10.1155/2023/6384208

**Published:** 2023-12-21

**Authors:** Amal F. Makled, Sahar A. M. Ali, S. S. Eldahdouh, Asmaa S. Sleem, Maha M. Eldahshan, Yara Elsaadawy, Samar S. Salman, Asmaa Mohammed Elbrolosy

**Affiliations:** ^1^Department of Medical Microbiology and Immunology, Faculty of Medicine, Menoufia University, Shebin al Kom, Egypt; ^2^Department of Chest Diseases and Tuberculosis, Faculty of Medicine, Menoufia University, Shebin al Kom, Egypt; ^3^Department of Medical Microbiology and Immunology, Faculty of Medicine, Ain Shams University, Cairo, Egypt; ^4^Department of Clinical Pathology, Faculty of Medicine, Menoufia University, Shebin al Kom, Egypt

## Abstract

**Introduction:**

The host genetic background is a crucial factor that underlies the interindividual variability of COVID-19 fatality and outcomes. Angiotensin-converting enzyme-2 (*ACE-2*) and interferon-induced transmembrane protein-3 (*IFITM-3*) have a key role in viral cell entrance and priming. The evoked immune response will also provide a predictive prognosis for COVID-19 infection. This study aimed to explore the association between *ACE-2* and *IFITM-3* genotypes and their corresponding allele frequencies with disease severity indices in the Egyptian COVID-19 population. The serum level of interleukin-6, as a biomarker of hyperinflammatory response, and cytokine storm, was correlated with disease progression, single nucleotide polymorphisms (SNPs) of the selected receptors, and treatment response. *Methodology*. We enrolled 900 COVID-19-confirmed cases and 100 healthy controls. Genomic DNA was extracted from 200 subjects (160 patients selected based on clinical and laboratory data and 40 healthy controls). The *ACE-2* rs2285666 and *IFITM-3* rs12252 SNPs were genotyped using the TaqMan probe allelic discrimination assay, and the serum IL-6 level was determined by ELISA. Logistic regression analysis was applied to analyze the association between *ACE-2* and *IFITM-3* genetic variants, IL-6 profile, and COVID-19 severity.

**Results:**

The identified genotypes and their alleles were significantly correlated with COVID-19 clinical deterioration as follows: *ACE2* rs2285666 CT + TT, odds ratio (95% confidence interval): 12.136 (2.784–52.896) and *IFITM-3* rs12252 AG + GG: 17.276 (3.673–81.249), both *p* < 0.001. Compared to the controls, the heterozygous and mutant genotypes for both SNPs were considerable risk factors for increased susceptibility to COVID-19. IL-6 levels were significantly correlated with disease progression (*p* < 0.001).

**Conclusion:**

*ACE-2* and *IFITM-3* genetic variants are potential predictors of COVID-19 severity, critical outcomes, and post-COVID-19 complications. Together, these SNPs and serum IL-6 levels explain a large proportion of the variability in the severity of COVID-19 infection and its consequences among Egyptian subjects.

## 1. Introduction

A novel pathogen called SARS-CoV-2 caused a viral respiratory infection known as the 2019 coronavirus disease (COVID-19) that spread across the globe. Approximately, 20% of the COVID-19 patients experience either severe pneumonia or a critical illness that results in death although the majority of the patients are asymptomatic or show mild sickness [1].

Host characteristics such as sex, concomitant comorbidities, ethnicity, and age group are ultimately related to the observed variation in clinical presentation and consequences of SARS-CoV-2 infection [2]. Recent research suggests that the genetic makeup of the host may also be a factor in the disparity between COVID-19 progression and case fatality rates [3].

Given their important roles in SARS-CoV-2 tropism to host cells, cell membrane binding, cell entry, replication, and immune response to the virus, a number of gene candidates were examined in this regard, including angiotensin-converting enzyme-2 (*ACE-2*), transmembrane serine protease-2 (*TMPRSS2*), interferon-induced transmembrane protein-3 (*IFITM-3*), toll-like receptor-3 (*TLR3*), and interferon regulatory factor-7 (*IRF-7*) [4].

The primary host cell receptor for the SARS-CoV-2 viral spike glycoprotein is *ACE-2* [5]. It enhances the entry of the virus into the cell, causing the final infection [6, 7], and alters host susceptibility to SARS-CoV-2, reflecting *ACE-2* spike glycoprotein interaction [8]. Genetic polymorphisms that control *ACE-2* expression have the potential to significantly alter COVID-19 severity and host cell response [9, 10]. Several SNPs were investigated in this context, including rs182366225, rs2097723, rs1027571965, and rs2285666. The majority of variations have been shown to be linked to *ACE-2* overexpression [11].

The IFITM family plays a pivotal role in the pathogenesis of many viral diseases. The *IFITM-3* gene encodes an interferon-induced membrane protein with broad antiviral activity against influenza A, H1N1 virus, and SARS-CoV-2. They mediate an innate immune response by regulating the fusion of the invading virus and endocytic vesicles directing it to lysosomes. *IFITM-3* can further alter membrane rigidity and curvature to prevent virus membrane fusion. Such action is required to inhibit the release of viral particles into the cytoplasm, thus controlling viral propagation [12]. Variants in the *IFITM-3* gene have been consistently associated with changes in expression and the risk of severe COVID-19 disease including rs6598045 [13], rs34481144 [14], and rs12252 [1].

The immunopathogenesis of COVID-19 is mostly influenced by the host inflammatory response and secreted cytokines, in addition to genetic differences. Despite varying degrees of evidence, the development of a cytokine storm led to the clinical form of COVID-19 infection deteriorating from mild to severe. Such a phenomenon has raised the question of whether exaggerated immunological sequelae can determine the severity of symptoms and contribute to a poorer outcome among COVID-19 patients [15].

Cytokine storm is an interesting point in SARS-CoV-2 infection. COVID-19 patients have elevated levels of inflammatory cytokines, particularly IL-6 [16, 17]. Furthermore, the potential predictive significance of IL-6 in terms of the need for mechanical ventilation or death has been confirmed [18]. Various existing medications can block the IL-6 pathway; however, only tocilizumab (an IL-6 receptor antagonist) has had a reasonable effect in COVID-19 [19].

Likewise, this study was conducted to increase the understanding of how the genetic background influences the severity of SARS-CoV-2 infection. We investigated the relationship between COVID-19 progression and genetic variants in both *ACE-2* and *IFITM-3*. We perhaps offer a strategy for identifying potential SNPs as prognostic markers with a particular attention to concurrent pandemic waves, serum IL-6 levels, clinical criteria, laboratory investigations, and treatment response among COVID-19 Egyptian population.

## 2. Subjects and Methods

### 2.1. Study Eligibility and Design

This case-control study was carried out at the Medical Microbiology and Immunology Department in collaboration with the Chest Department and Central Laboratory of the Faculty of Medicine, Menoufia University Hospitals (MUHs).

#### 2.1.1. Inclusion Criteria

- Age ≥18 years
- Positive laboratory findings or positive radiological findings specific to COVID-19
- Positive RT-PCR for COVID-19
- Healthy subjects who had no clinical findings suggesting any disease served as a control group

#### 2.1.2. Exclusion Criteria

- Patients without full laboratory and radiological work up
- Patients with negative RT-PCR findings for COVID-19
- Patients with a proved other concurrent acute illness

A total of 900 (539 males and 361 females) COVID-19-confirmed cases were recruited from ICUs and Chest Quarantine Wards of MUHs. Cases were arranged into the following two groups: group I (*n* = 380) collected during the third pandemic wave of SARS-CoV-2 in Egypt from the 1st of June to the end of August 2021 and group II (*n* = 520) collected during the fourth pandemic wave from the 1st of September to the end of November 2021. In addition, a control group of 100 healthy volunteers was included.

### 2.2. Clinical and Radiologic Assessments and Patients' Grouping

All patients underwent clinical evaluation in accordance with the WHO COVID-19 severity recommendations [20], and they were arranged into the following categories: adults with clinically evident pneumonia (fever, cough, dyspnea, and rapid breathing) but no symptoms of severity, such as peripheral oxygen saturation (SpO_2_) ≥90% on room air, were the subjects of mild and moderate cases (*n* = 200). Adults with clinical indications of pneumonia plus one of the following were included in severe cases (*n* = 360): respiration rate greater than 30 breaths per minute, acute respiratory distress, and room air SpO_2_ < 90%. Acute respiratory distress syndrome (ARDS), sepsis, and/or septic shock were among the critical cases (*n* = 340) (Tables S1 and S2).

Senior radiologists at MUHs used the Reporting and Data System (CO-RADS), a categorical assessment scheme for lung involvement in COVID-19, to undertake radiologic examination to estimate the severity of COVID-19. It involves a chest high-resolution CT, which on a scale from 1 (extremely low) to 6 (very high) predicts COVID-19 in patients with moderate to severe symptoms quite well [21].

### 2.3. Patients' Data Collection

Senior clinicians reviewed the medical records of the enrolled patients to gather information on their demographics, preexisting comorbidities, presenting symptoms, laboratory results (Hb, white blood cells (WBCs) count, platelets count, lymphocytes, CRP, D dimer, ferritin, LDH, AST, ALT, BUN, and creatinine serum levels), treatment regimens and response, complications, and expected outcomes. Daily data on the disease stage, the presence of fever, SpO_2_, partial pressure of oxygen in arterial blood (PaO_2_), and the necessity for invasive or noninvasive ventilator assistance were checked at the baseline and during hospitalization.

### 2.4. Patients' Follow-Up

The term “post-COVID-19 syndrome” describes symptoms that may be brought on by ongoing inflammation, organ damage, general hospital side effects, protracted ventilator support (postintensive care syndrome), or the impacts of underlying medical disorders [22]. After discharge, convalescent COVID-19 patients were assessed and monitored for up to 2 months for the occurrence of post-COVID-19 syndrome and the most prominent manifestations.

#### 2.4.1. Cases Selection

Because of limited financial support, two hundred subjects, i.e., 160 cases (40 mild/moderate, 60 severe, and 60 critical) and 40 healthy controls were selected based on clinical, laboratory, and radiological findings and subjected to genotypic analysis of both *ACE-2* (rs2285666) and *IFITM-3* (rs12252) SNP variants as well as serum IL-6 profiling as follows.

### 2.5. Blood Sample Collection and Preparation

A septic venipuncture was used to obtain 5 ml of venous blood and was processed as follows:

- 3 ml of blood were placed into the EDTA-containing tube for DNA extraction and genotyping of *ACE-2* (rs2285666) and *IFITM-3* (rs12252) SNPs by using the TaqMan allelic discrimination assay technique.
- The remaining 2 ml of blood was transferred into the plain tube, left to clot for 15 min, and centrifuged for 10 min at 4000 r.p.m. The serum obtained was stored at −80°C until analysis of serum IL-6 by ELISA.

### 2.6. Molecular Study

#### 2.6.1. Genomic DNA Extraction

Genomic DNA was extracted following the manufacturer's instructions using a GeneJET Whole Blood Genomic DNA Purification Mini Kit (Thermo Scientific, EU/Lithuania).

#### 2.6.2. Genotyping Assay

**Figure 1:**
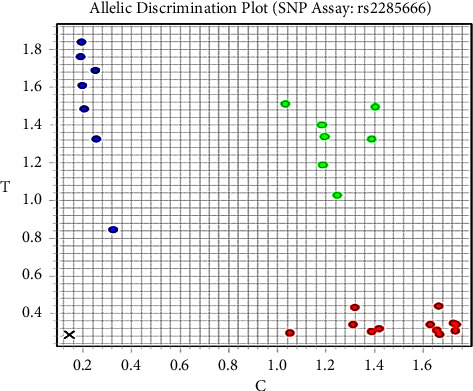
SNP genotyping scatter plot of *ACE2* (rs2285666). CC is the homozygous wild genotype (red dots), TT is the homozygous mutant genotype (blue dots), and CT is the heterozygous genotype (green dots).

**Figure 2:**
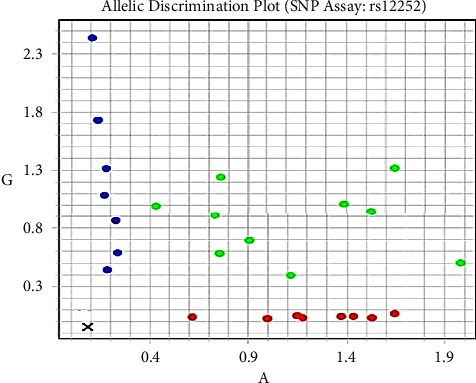
SNP genotyping scatter plot of *IFITM3* (rs12252). AA is the homozygous wild genotype (red dots), GG is the homozygous mutant genotype (blue dots), and AG is the heterozygous genotype (green dots).

The real-time polymerase chain reaction allelic discrimination technique was used to investigate the rs2285666 of *ACE-2* and the rs12252 of *IFITM-3* polymorphisms using the TaqMan SNP genotyping assay kit (Thermo Fisher Scientific, Waltham, MA, United States) with catalog no. C___2551626_1 and C_175677529_10, respectively, and their context sequences were as follows: (VIC/FAM) ATAATCACTACTAAAAATTAGTAGC (C/T) TACCTGGTTCAAGTAATAAGCATTC and (VIC/FAM) GCATCTCATAGTTGGGGGGCCTGG (A/G) CTGTTGACAGGAGAGAAGAAGGTT, respectively, as shown in Figures 1 and 2. The PCR mixture contained 7.5 *μ*l of TaqMan genotyping master mix (Applied Biosystems), 0.75 *μ*l of TaqMan SNP (probes), 1 *μ*l of genomic DNA (1–10 ng), and 5.75 *μ*l nuclease-free water. Thermal conditions were as follows: initial denaturation at 95°C for 10 min and 40 cycles were run at 95°C for 15 s (denaturing), followed by 60°C for 1 min (annealing/extension).

### 2.7. Interleukin-6 Serum Levels

Before undergoing anti-IL-6 therapy, the 1st reading of serum IL-6 levels was determined using an enzyme-linked immunosorbent assay with a human IL-6 ELISA kit (Chemux Bio Science, Inc., USA) in accordance with the manufacturer's instructions. Kinetic measurements of serum IL-6 (follow-up reading) were recorded for forty-six patients who received cilizumab (Actemra 20 mg/ml, Roche) as anti-IL-6 therapy 72 hours after treatment initiation at a dose of 4–8 mg/kg/d I.V. for 2 doses 12–24 h apart.

### 2.8. Statistical Analysis

The distribution of the variables' variance was assessed for normality. Nonparametric continuous variables were compared using the Kruskal‒Wallis test between the groups that were examined, and the results were summarized using means, medians, and interquartile ranges (IQRs). The Chi squared test was used for categorical variables. A value of *p* < 0.05 was regarded as statistically significant for all the tests. The association among genetic variations, IL-6, and other various parameters with COVID-19 susceptibility and outcomes was examined using a logistic regression approach. By analyzing genotype distribution using the Kruskal‒Wallis test and stratifying by disease outcome, it was possible to determine the relationship between SNPs and clinical characteristics. STATA v.13 statistical software tools (StataCorp, TX, USA) were used to carry out the analysis.

## 3. Results

### 3.1. Sociodemographic Data, Associated Comorbidities, and Clinical Presentation of the Studied Subjects

Male predominance was noticed among both studied groups, but the difference was not statistically significant. Meanwhile, the difference was highly significant for age distribution (*p* < 0.001). Smoking and the majority of underlying comorbidities were found to significantly differ across the groups under study (*p* < 0.001), as shown in Table 1. Fever, cough, and dyspnea were more prominent symptoms in group I than in group II (75.8% vs. 62.3%, 72.1% vs. 46.2%, and 66.3% vs. 55.6%, respectively), and the differences were highly significant (*p* ≤ 0.001). Moreover, the development of ARDS and sepsis was higher during the third pandemic wave (52.6% vs. 34.8% and 23.7% vs. 2.5%, respectively) (Table 2).

### 3.2. The Relation between COVID-19 Severity and Patients' Outcomes

ICU admission was more frequently recorded among patients in the third pandemic wave than among those in the fourth one (73.7% vs. 47.3%). In addition, the death rate was higher among patients in the third wave than among those in the fourth wave (55.3% vs. 44.2%), while recovery and post-COVID-19 sequelae were significantly more common during the fourth wave (52.1% vs. 34.2% and 34.2% vs. 24.7%, respectively), as shown in Table 3.

### 3.3. Genotypes and Allelic Distribution of Both *ACE-2* (rs2285666) and *IFITM-3* (rs12252) Polymorphisms

Importantly, we observed that both heterozygous CT and mutant TT genotypes of rs2285666 were more prevalent among severe (26.7% and 53.3%, respectively) and critical (33.3% and 53.3%, respectively) cases compared to either mild/moderate cases (15% and 10%, respectively) or the control group (20% and 5%, respectively), with a significant difference (*p*_1_ < 0.001, *p*_2_<0.001, and *p*_0_ < 0.001). In addition, the T allele displayed a pattern of 70%, 66.7%, and 17.5% for critical, severe, and mild/moderate cases, respectively. The mutant genotype GG for rs12252 of *IFITM-3* was predominant among severe (60.0%) and critical (41.7%) cases compared to both mild/moderate cases and controls (5% for each). A parallel distribution was also detected for the G allele, with higher percentages among severe (70.0%) and critical cases (61.7%). On the other hand, no statistically significant difference was noticed between the mild/moderate and control groups regarding genotypes and allelic frequencies of both SNPs (*p* > 0.05), as shown in Table 4. The dominant, recessive, codominant, and overdominant models for genotype combinations for both SNPs are shown in Tables S8 and S9.

### 3.4. Relationship between *ACE-2* (rs2285666) and *IFITM-3* (rs12252) Genotypes and Clinical Data in the Studied Patients

Patients carrying the CT and TT genotypes of rs2285666 suffered significantly more from dyspnea, ARDS, and sepsis than those carrying the wild-type CC genotype (dyspnea: 66.7% and 70.6% vs. 40%, ARDS: 42.9% and 58.8% vs. 20%, and sepsis: 4.8% and 23.5% vs. 4%). The CT and TT genotypes resulted in more deleterious outcomes, with 66.7% and 67.6% ICU admission and 47.6% and 67.6% death rates, respectively; however, the wild-type CC variant provided a 76% recovery rate with 66% liability for post-COVID-19 manifestations (Table S6). Similarly, the heterozygous AG and mutant GG variants of rs12252 were correlated with poorer consequences among the studied cases as well. Dyspnea and ARDS were common among patients with AG and GG variants compared to the reference AA genotype (60.9% and 74.6% vs. 41.2% for dyspnea and 47.8% and 52.4% vs. 25.5% for ARDS, respectively). Even ICU admission and mortality rates were recorded for AG and GG genotypes by 65.2% and 68.3% and 56.5% and 65.1%, respectively, which was highly statistically significant (*p* < 0.001). The recovery rate reached 78.4% for the AA genotype compared to only 32.6% and 27% for the hetero and mutant genotypes, respectively, as illustrated in Table S7.

### 3.5. Serum Level of IL-6 (1^st^ Reading) among the Selected Participants (*n* = 200) from Both Pandemic Waves

As shown in Table 5, the serum IL-6 profile denoted higher levels along with increasing disease severity and clinical deterioration, with a highly significant difference among the studied cases (*p* < 0.001).

### 3.6. Interleukin-6 Levels before and after Receiving Anti-IL-6 Treatment According to Group Severity (*n* = 46)

The follow-up was performed by measuring IL-6 levels after receiving anti-IL-6 therapy for 46 patients. The mean value of IL-6 decreased from 48.77 ± 66.20 to 21.88 ± 33.18 pg/ml (*p*=0.003) for mild/moderate cases. Surprisingly, ongoing higher IL-6 levels were detected for severe and critical cases even after receiving an anti-IL-6 regimen, denoting the development of a cytokine storm (Table 6).

### 3.7. Logistic Regression Analysis for Predictors of COVID-19 Severity and Susceptibility

*IFITM-3* AG + GG, *ACE-2* CT + TT variants, and IL-6 were found to be significant predictors for COVID-19 severity (*p* < 0.001) in a univariate logistic regression analysis. Moreover, the multivariate analysis demonstrated that *ACE-2* CT + TT, *IFITM-3* AG + GG variants, and IL-6 were independent risk factors for COVID-19 severity and clinical progression with odds ratios (95% C. I) of 12.136 (2.784–52.896), 17.276 (3.673–81.249), and 1.032 (1.012–1.052) (*p* = 0.001, *p* < 0.010, and *p* = 0.002), respectively. In addition, CRP, D-dimer, ferritin, associated comorbidities, and age were significant predictors of severity in univariate analysis. Regarding COVID-19 susceptibility, the analyzed data revealed that both *ACE-2* CT+TT and *IFITM-3* AG+GG genotypes significantly correlated to COVID-19 susceptibility, as shown in Tables 7 and 8.

## 4. Discussion

Accompanying the spread of COVID-19 disease all over the world, recognizing factors connected to the susceptibility and outcome of the disease is at the top of medical and pharmacological concerns [23]. This work was performed to identify possible COVID-19 prognostic markers with special concern for *ACE-2* and *IFITM-3* genetic variations and IL-6 level assays.

COVID-19 susceptibility and severity among studied cases were linked to age and associated comorbidities. Nikkhoo et al. documented the same finding [24, 25].

The diseased patients' outcome in our study showed a significant decline in clinical and laboratory conditions in the third pandemic wave compared to the fourth one, especially for ICU hospitalization and death records. Such observations were also reported in Iran by Amin et al. on COVID-19 consecutive waves with increased severity and mortality during the early three waves compared to followers [26].

Laboratory data from our patients and findings from further research revealed that patients with severe and critical illness had considerably higher levels of CRP, D-dimer, and serum ferritin compared to mild/moderate cases (Tables S1 and S2) [27, 28]. In addition, Gómez-Haranz et al. reported significant correlations between case severity and different laboratory markers, including CRP, D-dimer, and ferritin levels [29]. Fever, coughing, and dyspnea were mostly the prominent presentations in patients during the third pandemic wave. These observations were supported by other studies [30–32].

COVID-19-infected patients can develop variable illnesses with unexpected consequences. Although many disease predictors have been reported, much research is still needed. Infection rates and outcomes could be dependent on the host's genetic background, especially those affecting the virus-cell interaction [27]. The main finding in this study was the higher prevalence of hetero and mutant genotypes of *ACE-2* rs2285666 among severe (CT 26.7% and TT 53.3%) and critical (CT 33.3% and TT 53.3%) cases than that of controls (CT 15.0% and TT 10%). These genotypes were risk factors for COVID-19 severity and enhanced susceptibility according to logistic regression analysis (Tables 7). Likewise, the T allele displayed the same pattern, with 66.7% and 70% predominance among severe and critical cases, respectively. These data were similar to recent data reported by Abdel Sattar et al. in Egypt, who found that the genotypes CT and TT and the T allele were high predictors of severe illness [28]. In contrast, Sidhawani et al. in Pakistan found that the TT genotype of rs2285666 was protective, while the CC genotype raised the likelihood of COVID-19 disease [27]. Çelik et al. did not find any link between the rs2285666 polymorphism and disease severity [33].

The *ACE-2* rs2285666 variant is located in the third intron of the gene; thus, the expression of the gene is affected by an alternative splicing mechanism [34]. According to Asselta et al., the change from C to T strengthened the splicing site by 9.2%, leading to enhanced production of the *ACE-2* protein [35]. In addition, Gemmati and Tisato declared the *ACE-2* gene as a “first genetic gateway” involved in COVID-19 infection and severity [36].

A study by Martinez Gómez et al. showed that when the T allele of rs2285666 was present, the risk for developing severe and critical consequences in COVID-19 was increased, especially in men. The authors also declared a link between the TT genotype of rs2285666 and an increased likelihood of more serious sequelae with SARS-CoV-2 infection [37]. Furthermore, Benetti et al. showed that *ACE-2* genetic variations affect protein function and may result in differing susceptibility and progression of COVID-19 infection [38]. Srivastava et al. stated that genetic differences in ACE-2 affect susceptibility to COVID-19, and carriers of the T allele had a decreased infection rate in Indian populations [39]. However, it is crucial to note that several variables contribute to SARS-CoV-2 infection, and *ACE-2* may not be the sole gene involved. Ethnic differences in populations as well as variations in gene expression could be responsible for these discrepancies [37].

Endogenous expression of *IFITM-3* was shown to be necessary for the replication of numerous coronaviruses as previously mentioned by Xie and colleagues and Wang and colleagues [40, 41]. The altered expression of *IFITM-3* changes the cellular expression and localization of *ACE-2* with subsequent opposing or enhancing unclear impacts on virus entry [42]. According to their reports, *IFITM-3* rs12252 AG and GG polymorphic variants were predominant in severe and critical groups, which could be explained by enhanced *ACE-2* binding availability. These data coincided with our findings, where *ACE-2* (CT + TT) and *IFITM-3* (AG + GG) SNP associations were the best indicators of illness severity by logistic regression analysis (Table 6). Our study was in line with that by Zhang et al., where the rising frequency of genotype GG for rs12252 was reported among severe cases. A meta-analytic study involving more than four studies from England, Italy, and Spain showed a significant association of the *IFITM-3* rs12252/*ACE-2* rs2285666 polymorphism with COVID-19 susceptibility [43]. However, neither rs2285666 nor rs12252 were shown to be substantially related to illness severity [23].

The mechanisms by which *IFITM-3* rs12252 with the G allele is associated with COVID-19 deterioration are not completely understood. The variation is thought to mediate lowered *IFITM-3* protein expression and, as a result, weakened the antiviral activity due to truncation of the encoded 21-amino acid or alteration of the protein's cellular localization between the membrane of the cell and endosomal vesicles [44–46].

The current study explored IL-6 as a disease progression marker owing to its characteristic role in the COVID-19-associated cytokine storm, reflecting its importance as a pharmacological target. Our statistics demonstrated that elevated serum IL-6 levels were significantly correlated with severe and critical illness (*p* < 0.001). Many studies have been conducted to examine the predictive usefulness of IL-6 on various clinical features of COVID-19 infection with high specificity [19, 47]. Furthermore, IL-6 levels were found to be substantially related to various clinical and laboratory indicators predicting a systemic inflammatory response, such as CRP, ferritin, D-dimer, and LDH (Table S3). The same correlation was reported by other studies; however, IL-6 seemed to be the major prognostic effector [24, 47].

IL-6 levels together with *ACE-2* rs2285666 and *IFITM-3* rs12252 genetic variants were suggested here as significant predictors for COVID-19 fatality. Interestingly, we observed higher serum IL-6 levels among patients harbouring the CT variant compared to the wild CC type of rs2285666, as provided in the supplementary files (Table S4). However, Beyranvand et al. did not find any correlation between IL-6 levels and SNP polymorphisms of the two target receptors [48, 49].

IL-6 inhibitors are strongly recommended by the WHO and National Institute for Health and Care Excellence (NICE) in COVID-19 patients either prescribed alone or with corticosteroids [50]. The studies implemented by Sciascia et al. [51, 52] demonstrated an obvious improvement among most patients who received tocilizumab (TCZ), as proven in our results, particularly for mild/moderate cases (Table 5). In addition, Salvati et al. declared that the administration of TCZ increased the likelihood of survival among moderate cases [53]. Similar to our results, Hermine et al. documented that in critically ill adult patients with COVID-19 pneumonia, anti-IL-6 receptor therapy did not improve early survival in the absence of mechanical respiration [54]. Perhaps, the cytokine storm and shooting of the IL-6 levels occurring in both severe and critical cases hindered the effect of TCZ. Contrary to our results, Shankar-Hari and colleagues demonstrated a clinical benefit (84.9%) with the use of TCZ in COVID-19 patients requiring organ support with hypoxemia and/or systemic inflammation [55].

The majority of people who have COVID-19 recover completely; however, approximately 10–20% develops a variety of mid- and long-term sequelae after recovering from their original sickness [56]. According to our results, patients in different severity groups experienced post-COVID-19 symptoms in variable proportions. Carvalho-Schneider et al. [57, 58] documented the occurrence of dyspnea and fatigue in 30% and 40% of COVID-19 patients, respectively, during their follow-up study, which partially matches our results showing dyspnea and fatigue being persistent in 30% and 20%, and 40% and 30% of patients within the third and fourth waves, respectively (Table S5).

Approximately, 80% of the noncritical patients with COVID-19 recovered completely, and death was recorded only among the critical cases [59]. These findings were in line with our results. In a study conducted by Abdelhafiz et al., the prevalence of post-COVID-19 symptoms was 87.63% and fatigue was the most frequent symptom (60.86%) [60].

## 5. Conclusion

Both *ACE-2* and *IFITM-3* SNPs were amongst the highest determinants of COVID-19 severity, together with IL-6, CRP, D-dimer, ferritin, preexisting comorbidities, and age. IL-6 is a prognostic and therapeutic target for COVID-19 patients. Both SNPs and serum IL-6 levels could explain a large proportion of variability in the susceptibility and severity of COVID-19 infection and outcome among Egyptian subjects.

### 5.1. Study Limitation

We missed recording some early signs and symptoms of admitted patients due to difficult communications with the patients, particularly those admitted immediately with severe and critical forms of the disease and thus relied solely on their recorded data.

There was difficulty in performing genotyping and interleukin-6 ELISA level analysis for more than two hundred participants due to financial issue.

## Figures and Tables

**Table 1:** Comparison between the studied populations regarding sociodemographic data and associated comorbidities.

	Group I patients in wave 3 (*n* = 380)		Group II patients in wave 4 (*n* = 520)		Control (*n* = 100)		*p*
	No.	%	No.	%	No.	%	
Gender							
Male	238	62.6	301	57.9	59	59.0	0.352
Female	142	37.4	219	42.1	41	41.0	
Age (years)							
Min.–Max.	24.0–80.0		19.0–84.0		33.0–77.0		<0.001^*∗*^
Mean ± SD	62.85 ± 14.56		56.74 ± 14.94		58.26 ± 15.18		
Sig. bet. grps.	*p*_1_ < 0.001^*∗*^, *p*_2_=0.016^*∗*^, *p*_3_=0.616						
Smoking	76	20.0	64	12.3	20	20.0	0.004^*∗*^
Obesity	20	5.3	40	7.7	5	5.0	0.280
Pregnancy	18	4.7	38	7.3	0	0.0	0.009^*∗*^
Comorbidities							
DM	146	38.4	183	35.2	13	13.0	<0.001^*∗*^
Sig. bet. grps.	*p*_1_=0.321, *p*_2_ < 0.001^*∗*^, *p*_3_ < 0.001^*∗*^						
HTN	196	51.6	207	39.8	17	17.0	<0.001^*∗*^
Sig. bet. grps.	*p*_1_ < 0.001^*∗*^, *p*_2_ < 0.001^*∗*^, *p*_3_ < 0.001^*∗*^						
COPD	96	25.3	90	17.3	8	8.0	<0.001^*∗*^
Sig. bet. grps.	*p*_1_=0.004^*∗*^, *p*_2_ < 0.001^*∗*^, *p*_3_=0.019^*∗*^						
CHD	140	36.8	101	19.4	5	5.0	<0.001^*∗*^
Sig. bet. grps.	*p*_1_ < 0.001^*∗*^, *p*_2_ < 0.001^*∗*^, *p*_3_ < 0.001^*∗*^						
CKD	110	28.9	101	19.4	3	3.0	<0.001^*∗*^
Sig. bet. grps.	*p*_1_=0.001^*∗*^, *p*_2_ < 0.001^*∗*^, *p*_3_ < 0.001^*∗*^						
CLD	30	7.9	63	12.1	6	6.0	0.043^*∗*^
Sig. bet. grps.	*p*_1_=0.040^*∗*^, *p*_2_=0.522, *p*_3_=0.075						
Carcinoma	10	2.6	13	2.5	0	0.0	0.268
Vaccine	0	0.0	25	4.8	30	30.0	<0.001^*∗*^

Group I: patients of the third COVID-19 wave, group II: patients of the fourth COVID-19 wave, and SD: standard deviation. *p*: *p* value for comparing between the three studied groups. Sig. bet. grps: *p* value for comparing between group I and group II and for comparing between both groups and control as follows: *p*_1_: *p* value for comparing between group I and group II. *p*_2_: *p* value for comparing between group I and controls. *p*_3_: *p* value for comparing between group II and controls. ^*∗*^Statistically significant at *p* ≤ 0.05. DM: diabetes mellitus, HTN: hypertension, COPD: chronic obstructive pulmonary disease, CHD: chronic heart disease, CKD: chronic kidney disease, and CLD: chronic liver disease.

**Table 2:** Comparison between the studied populations regarding clinical presentation and complications.

	Group I (*n* = 380)		Group II (*n* = 520)		*p*
	No.	%	No.	%	
*Clinical presentation*					
Fever	288	75.8	324	62.3	<0.001^*∗*^
Cough	274	72.1	240	46.2	<0.001^*∗*^
Dyspnea	252	66.3	289	55.6	0.001^*∗*^
Headache	46	12.1	78	15.0	0.213
Chest pain	38	10.0	51	9.8	0.924
Vomiting or nausea	38	10.0	51	9.8	0.924
Diarrhea	38	10.0	51	9.8	0.924
Myalgia or arthralgia	90	23.7	119	22.9	0.779
Disturbed conscious level	60	15.8	91	17.5	0.498

*Complications*					
ARDS	200	52.6	181	34.8	<0.001^*∗*^
AKI	110	28.9	142	27.3	0.588
Sepsis	90	23.7	13	2.5	<0.001^*∗*^

*Recurrence*	20	5.3	53	10.2	0.007^*∗*^

*p*: *p* value for comparing between the two studied groups. ^*∗*^Statistically significant at *p* ≤ 0.05. ARDS: acute respiratory distress syndrome and AKI: acute kidney injury.

**Table 3:** Relation between COVID-19 severity and patients' outcomes among the studied cases of wave 3 and 4.

Outcomes		Mild/moderate		Severe		Critical			Total	*p*_0_
		No.	%	No.	%	No.		%		
		(*n* = 80)		(*n* = 150)		(*n* = 150)				
Group I	Recovery	80	100.0	50	33.3	0	0.0		130 (34.2%)	<0.001^*∗*^
	ICU admission	0	0.0	130	86.7	150	100.0		280 (73.7%)	<0.001^*∗*^
	Death (nonsurvivors)	0	0.0	85	56.7	125	83.3		210 (55.3%)	<0.001^*∗*^
	Post-COVID-19 syndrome	64	80.0	30	20.0	0	0.0		94 (24.7%)	<0.001^*∗*^

		(*n* = 120)		(*n* = 210)		(*n* = 190)				

Group II	Recovery	120	100.0	112	53.3	39	20.5		271 (52.1%)	<0.001^*∗*^
	ICU admission	0	0.0	56	26.7	190	100.0		246 (47.3%)	<0.001^*∗*^
	Death (nonsurvivors)	0	0.0	98	46.7	132	69.5		230 (44.2%)	<0.001^*∗*^
	Post-COVID-19 syndrome	96	80.0	57	27.1	25	13.2		178 (34.2%)	<0.001^*∗*^

Sig. bet. grps.		*p*_1_ < 0.001^*∗*^, *p*_2_ < 0.001^*∗*^, *p*_3_ < 0.001^*∗*^, *p*_4_=0.002^*∗*^								

*p*_0_: *p* value for the Chi square test for comparing between mild/moderate and severe and critical in both groups. Sig. bet. grps: *p* value for comparing between group I and group II regarding different outcomes parameters (total) as follows: *p*_1_: *p* value for comparing between group I and group II regarding total recovery. *p*_2_: *p* value for comparing between group I and group II regarding total ICU admission. *p*_3_: *p* value for comparing between group I and group II regarding total death. *p*_4_: *p* value for comparing between group I and group II regarding total post-COVID-19 syndrome. ^*∗*^Statistically significant at *p* ≤ 0.05.

**Table 4:** Genotypes and allelic frequencies of *ACE-2* (rs2285666) and *IFITM-3* (rs12252) among the studied cases regarding COVID-19 severity.

SNPs	Mild/moderate (*n* = 40)		Severe (*n* = 60)		Critical (*n* = 60)		Control (*n* = 40)		*p*
	No.	%	No.	%	No.	%	No.	%	
rs2285666									
Genotype									
C\C	30	75.0	12	20.0	8	13.3	30	75.0	<0.001^*∗*^
C\T	6	15.0	16	26.7	20	33.3	8	20.0	
T\T	4	10.0	32	53.3	32	53.3	2	5.0	
*p*_0_	^MC^ *p*=0.693		<0.001^*∗*^		<0.001^*∗*^				
Sig. bet. grps.	*p*_1_ < 0.001^*∗*^, *p*_2_ < 0.001^*∗*^, *p*_3_=0.537								
Allele									
C	66	82.5	40	33.3	36	30.0	68	85.0	<0.001^*∗*^
T	14	17.5	80	66.7	84	70.0	12	15.0	
*p*_0_	0.668		<0.001^*∗*^		<0.001^*∗*^				
Sig. bet. grps.	*p*_1_ < 0.001^*∗*^, *p*_2_ < 0.001^*∗*^, *p*_3_=0.579								
rs12252									
Genotype									
A\A	28	70.0	12	20.0	11	18.3	30	75.0	<0.001^*∗*^
A\G	10	25.0	12	20.0	24	40.0	8	20.0	
G\G	2	5.0	36	60.0	25	41.7	2	5.0	
*p*_0_	^MC^ *p*=0.910		<0.001^*∗*^		<0.001^*∗*^				
Sig. bet. grps.	*p*_1_ < 0.001^*∗*^, *p*_2_ < 0.001^*∗*^, *p*_3_=0.049^*∗*^								
Allele									
A	66	82.5	36	30.0	46	38.3	68	85.0	<0.001^*∗*^
G	14	17.5	84	70.0	74	61.7	12	15.0	
*p*_0_	0.668		<0.001^*∗*^		<0.001^*∗*^				
Sig. bet. grps.	*p*_1_ < 0.001^*∗*^, *p*_2_ < 0.001^*∗*^, *p*_3_=0.174								

C\C, A\A: wild genotype. A\G, C\T: hetero genotype. G\G, T\T: mutant genotype. MC: Monte Carlo. *χ*^2^: Chi square test. *p*_0_: *p* value for the Chi square test for comparing between control and each other groups. *p*_1_: *p* value for the Chi square test for comparing between mild/moderate and severe. *p*_2_: *p* value for the Chi square test for comparing between mild/moderate and critical. *p*_3_: *p* value for the Chi square test for comparing between severe and critical. ^*∗*^Statistically significant at *p* ≤ 0.05.

**Table 5:** Comparison between IL-6 serum 1^st^ reading among the selected participants (*n* = 200) of both pandemic waves.

IL-6 1^st^reading (pg\ml)	Mild/moderate (*n* = 40)	Severe (*n* = 60)	Critical (*n* = 60)	Control (*n* = 40)	*p*
Min.–Max.	2.45–224.0	3.0–484.0	8.0–531.0	1.0–6.0	<0.001^*∗*^
Mean ± SD.	22.60 ± 39.97	80.38 ± 102.8	135.8 ± 126.0	3.63 ± 1.39	
Median (IQR)	8.65 (5.0–18.0)	31.2 (16.0–116.0)	84.3 (35.0–200.0)	3.50 (3.0–5.0)	
*p*_0_	<0.001^*∗*^	<0.001^*∗*^	<0.001^*∗*^		
Sig. bet. subgrps.	*p*_1_<0.001^*∗*^, *p*_2_<0.001^*∗*^, *p*_3_ = 0.009^*∗*^				

IQR: interquartile range SD: standard deviation. *p*: *p* value for comparing between the four studied groups. *p*_0_: *p* value for comparing between control and each other groups. *p*_1_: *p* value for comparing between mild/moderate and severe. *p*_2_: *p* value for comparing between mild/moderate and critical. *p*_3_: *p* value for comparing between severe and critical. ^*∗*^Statistically significant at *p* ≤ 0.05.

**Table 6:** Comparison between IL-6 levels before and after receiving anti-IL-6 treatment regarding group severity (*n* = 46).

Cases	(*n* = 46)	IL-6 level (pg\ml)				*p*
		1^st^ reading		Follow-up reading		
		Mean ± SD.	Median (min.–max.)	Mean ± SD.	Median (min.–max.)	
Mild/moderate	11	48.77 ± 66.20	18.0 (8.65–224.0)	21.88 ± 33.18	8.0 (6.50–117.0)	0.003^*∗*^
Severe	12	60.25 ± 85.89	18.50 (5.0–237.0)	208.4 ± 257.4	79.0 (27.0–726.0)	0.002^*∗*^
Critical	23	146.0 ± 132.5	115.0 (16.30–531.0)	249.3 ± 151.4	243.0 (9.0–633.0)	<0.001^*∗*^

SD: standard deviation. *p*: *p* value for relation between 1^st^ reading and follow-up reading. ^*∗*^Statistically significant at *p* ≤ 0.05.

**Table 7:** Univariate and multivariate logistic regression analyses for predictors of COVID-19 progression among the studied cases: severe and critical vs. mild/moderate cases (*n* = 120 vs. 40).

Parameters	Univariate		^#^Multivariate	
	*p*	OR (LL–UL 95% CI)	*p*	OR (LL–UL 95% CI)
*ACE2* (rs2285666) (CT + TT)	<0.001^*∗*^	15.0 (6.336–35.511)	<0.001^*∗*^	12.136 (2.784–52.896)
*IFITM-3* (rs12252) (AG + GG)	<0.001^*∗*^	9.841 (4.357–22.23)	<0.001^*∗*^	17.276 (3.673–81.249)
IL-6 level (1^st^ reading)	<0.001^*∗*^	1.024 (1.011–1.037)	0.002^*∗*^	1.032 (1.012–1.052)
CRP	0.005^*∗*^	1.017 (1.005–1.030)	0.669	1.006 (0.980–1.032)
D-dimer	<0.001^*∗*^	1.003 (1.001–1.005)	0.089	1.003 (1.000–1.005)
Ferritin	<0.001^*∗*^	1.005 (1.003–1.007)	0.676	1.000 (0.998–1.002)
Lymphopenia	0.426	1.362 (0.636–2.917)		
Comorbidities	0.001^*∗*^	3.778 (1.686–8.467)	0.099	8.019 (0.674–95.44)
Age (years)	0.012^*∗*^	1.030 (1.007–1.054)	0.711	1.010 (0.956–1.068)
Female	1.000	1.000 (0.482–2.076)		

OR: odds ratio. CI: confidence interval. LL: lower limit. UL: upper limit. #All variables with *p* < 0.05 were included in the multivariate analysis. ^*∗*^Statistically significant at *p* ≤ 0.05.

**Table 8:** Univariate and multivariate logistic regression analyses for predictors of COVID-19 susceptibility: cases vs. controls (*n* = 160 vs. 40).

Parameters	Univariate		^#^Multivariate	
	*p*	OR (LL–UL 95% CI)	*p*	OR (LL–UL 95% CI)
*ACE2* (rs2285666) (CT + TT)	<0.001^*∗*^	6.600 (2.996–14.541)	0.617	1.760 (0.192–16.148)
*IFITM-3* (rs12252) (AG + GG)	<0.001^*∗*^	6.412 (2.912–14.116)	0.231	4.225 (0.399–44.725)
Comorbidities	<0.001^*∗*^	5.559 (2.659–11.620)	0.605	1.684 (0.233–12.167)
Age (years)	0.632	1.005 (0.983–1.028)		
Female	1.000	1.000 (0.493–2.028)		

OR: odds ratio. CI: confidence interval. LL: lower limit. UL: upper limit. ^#^All variables with *p* < 0.05 were included in the multivariate analysis. ^*∗*^Statistically significant at *p* ≤ 0.05.

## Data Availability

The data used to support the findings of this study are available from the corresponding author on request.

## Abbreviations

SNP: Single nucleotide polymorphism
ACE2: Angiotensin-converting enzyme-2
IFITM-3: Interferon-induced transmembrane protein-3
COPD: Chronic obstructive pulmonary disease
ARDS: Acute respiratory distress syndrome.

## Supplementary Materials

Supplementary Materials Supplementary Table 1: classification of the studied cases according to COVID-19 severity guidelines (patients in wave 3; *n* = 380). Supplementary Table 2: classification of the studied cases according to COVID-19 severity guidelines (patients in wave 4; *n* = 520). Supplementary Table 3: correlation between 1^st^ reading IL-6 levels and laboratory parameters in the selected patients (*n* = 160). Supplementary Table 4: relation between 1^st^ reading IL-6 levels and rs2285666\rs12252 genotypes and alleles in the studied patients (*n* = 160). Supplementary Table 5: relation between COVID-19 severity and the most prominent post-COVID-19 syndrome presentations among the two studied waves. Supplementary Table 6: relation between *ACE-2* (rs2285666) genotypes and clinical data in the studied patients (*n* = 160). Supplementary Table 7: relation between *IFITM-3* (rs12252) genotypes and clinical data in the studied patients (*n* = 160). Supplementary Table 8: comparison between the three studied groups and control according to rs2285666 in different genetic models. Supplementary Table 9: comparison between the three studied groups and control according to rs12252 in different genetic models.
