# Supplementary material for: Angiotensin-Converting Enzyme-2 (ACE-2) with Interferon-Induced Transmembrane Protein-3 (IFITM-3) Genetic Variants and Interleukin-6 as Severity and Risk Predictors among COVID-19 Egyptian Population
Source: Int J Microbiol. 2023 Dec 21;2023:6384208. doi: 10.1155/2023/6384208 (PMC10754637; doi:10.1155/2023/6384208)
Supplement: Supplementary Materials — Supplementary Table 1: classification of the studied cases according to COVID-19 severity guidelines (patients in wave 3; n = 380). Supplementary Table 2: classification of the studied cases according to COVID-19 severity guidelines (patients in wave 4; n = 520). Supplementary Table 3: correlation between 1st reading IL-6 levels and laboratory parameters in the selected patients (n = 160). Supplementary Table 4: relation between 1st reading IL-6 levels and rs2285666\rs12252 genotypes and alleles in the studied patients (n = 160). Supplementary Table 5: relation between COVID-19 severity and the most prominent post-COVID-19 syndrome presentations among the two studied waves. Supplementary Table 6: relation between ACE-2 (rs2285666) genotypes and clinical data in the studied patients (n = 160). Supplementary Table 7: relation between IFITM-3 (rs12252) genotypes and clinical data in the studied patients (n = 160). Supplementary Table 8: comparison between the three studied groups and control according to rs2285666 in different genetic models. Supplementary Table 9: comparison between the three studied groups and control according to rs12252 in different genetic models. [file 6384208.f1.docx]

**Supplementary tables**

**Supplementary Table 1: Classification of the studied cases according to COVID-19** **severity guidelines (patients in wave 3; n = 380)**

| **Severity indices** | **Mild/moderate (n = 80)** | | | **Severe (n = 150)** | | **Critical (n = 150)** | | | | **P** |
| --- | --- | --- | --- | --- | --- | --- | --- | --- | --- | --- |
|  | **No.** | **%** | | **No.** | **%** | **No.** | | | **%** |  |
| **Clinical presentation** |  |  | |  |  |  | | |  |  |
| Fever | 48 | 60.0 | | 130 | 86.7 | 110 | | | 73.3 | <0.001^*^ |
| Cough | 64 | 80.0 | | 100 | 66.7 | 110 | | | 73.3 | 0.091 |
| Dyspnea | 32 | 40.0 | | 110 | 73.3 | 110 | | | 73.3 | <0.001^*^ |
| **Complications** |  |  | |  |  |  | | |  |  |
| ARDS | 0 | 0.0 | | 90 | 60.0 | 110 | | | 73.3 | <0.001^*^ |
| AKI | 0 | 0.0 | | 60 | 40.0 | 50 | | | 33.3 | <0.001^*^ |
| Sepsis | 0 | 0.0 | | 50 | 33.3 | 40 | | | 26.7 | <0.001^*^ |
| **Oxygen saturation (%)** |  | | |  | |  | | | |  |
| Min. – Max. | 92.0 – 98.0 | | | 66.0 – 89.0 | | 65.0 – 88.0 | | | | <0.001^*^ |
| Mean ± SD. | 94.70±1.96 | | | 80.07±6.04 | | 76.60±6.82 | | | |  |
| **Sig. bet. grps.** | p_1_<0.001^*^,p_2_<0.001^*^,p_3_<0.001^*^ | | | | | | | | |  |
| **Respiratory rate** |  | | |  | |  | | | |  |
| Min. – Max. | 16.0 – 31.0 | | | 26.0 – 38.0 | | 27.0 – 36.0 | | | | <0.001^*^ |
| Mean ± SD. | 22.55 ± 4.32 | | | 31.0 ± 3.11 | | 32.0 ± 2.69 | | | |  |
| **Sig. bet. grps.** | p_1_<0.001^*^,p_2_<0.001^*^,p_3_=0.022^*^ | | | | | | | | |  |
| **CRP (mg/dL)** |  | | |  | |  | | | |  |
| Min. – Max. | 3.0 – 94.0 | | | 6.0 – 118.0 | | 4.0 – 240.0 | | | | <0.001^*^ |
| Median (IQR) | 24.0 (12.0 – 48.0) | | | 48.0 (24.0 – 96.0) | | 48.0 (24.0 – 115.0) | | | |  |
| **Sig. bet. grps.** | p_1_<0.001^*^,p_2_<0.001^*^,p_3_=0.442 | | | | | | | | |  |
| **Lymphocytes (10^3^cell/µL)** |  | | |  | |  | | | |  |
| Min. – Max.  Mean ± SD. | 0.70 – 2.90  1.33 ± 0.83 | | | 0.50 – 2.80  0.89 ± 0.58 | | 0.45 – 2.80  1.08 ± 0.82 | | | | 0.001^*^ |
| **Sig. bet. grps.** | p_1_=0.003^*^,p_2_=0.674,p_3_=0.002^*^ | | | | | | | | |  |
| **Ferritin(ng/mL)** |  | | |  | | |  | | | <0.001^*^ |
| Min. – Max. | 8.69 – 278.0 | | | 125.0 – 894.0 | | | 222.0 – 1990.0 | | |  |
| Median (IQR) | 162.5 (116.0 – 231.0) | | | 510.0 (311.0 – 660.0) | | | 686.0 (275.0 – 889.0) | | |  |
| **Sig. bet. grps.** | p_1_<0.001^*^,p_2_<0.001^*^,p_3_<0.001^*^ | | | | | | | | |  |
| **D-dimer(ng/mL)**  Min. – Max.  Median (IQR) | 0.43 – 345.0 | | 0.50 – 1619.0 | | | | | 0.90 – 984.0 | | <0.001^*^ |
|  | 5.45 (1.22 – 35.0) | | 146.0 (3.10 – 988.0) | | | | | 118.0 (1.70 – 432.0) | |  |
| **Sig. bet. grps.** | p_1_<0.001^*^,p_2_<0.001^*^,p_3_=0.122 | | | | | | | | |  |
| **Radiological CT** |  | | |  | | |  | | |  |
| CO-RADS3 | 40 (50.0%) | | | 30 (20.0%) | | | 10 (6.7%) | | | <0.001^*^ |
| CO-RADS4 | 40 (50.0%) | | | 80 (53.3%) | | | 60 (40.0%) | | |  |
| CO-RADS5 | 0 (0.0%) | | | 40 (26.7%) | | | 80 (53.3%) | | |  |

IQR: **Inter quartile range** SD: **Standard deviation**  χ^2^: **Chi square test**

H: H for **Kruskal Wallis test,** Pairwise comparison bet. each 2 groups was done using **Post Hoc Test (Dunn's for multiple comparisons test)**

p: p value for comparing between the three studied groups

p_1_: p value for comparing between **Mild/moderate** and **Severe**

p_2_: p value for comparing between **Mild/moderate** and **Critical**

p_3_: p value for comparing between **Severe** and **Critical**

*: Statistically significant at p ≤ 0.05 **^#^**: WHO, 2020 classification

**ARDS**: acute respiratory distress syndrome, **AKI**: acute kidney injury**, CRP**: C reactive protein **CO-RADS:** coronavirus disease 2019 (COVID-19) Reporting and Data System

**Supplementary Table 2:** **Classification of the studied cases according to COVID-19 severity guidelines (patients in wave 4; n = 520)**

|  | **Mild/moderate (n = 120)** | | **Severe (n = 210)** | | | | **Critical (n = 190)** | | **P** | |
| --- | --- | --- | --- | --- | --- | --- | --- | --- | --- | --- |
|  | **No.** | **%** | **No.** | | **%** | | **No.** | **%** |  |  |
| **Clinical presentation** |  |  |  | |  | |  |  |  | |
| Fever | 72 | 60.0 | 125 | | 59.5 | | 127 | 66.8 | 0.269 | |
| Cough | 48 | 40.0 | 128 | | 61.0 | | 64 | 33.7 | <0.001^*^ | |
| Dyspnea | 12 | 10.0 | 112 | | 53.3 | | 165 | 86.8 | <0.001^*^ | |
| **Complications** |  |  |  | |  | |  |  |  | |
| ARDS | 0 | 0.0 | 43 | | 20.5 | | 138 | 72.6 | <0.001^*^ | |
| AKI | 48 | 40.0 | 55 | | 26.2 | | 39 | 20.5 | <0.001^*^ | |
| Sepsis | 0 | 0.0 | 0 | | 0.0 | | 13 | 6.8 | ^MC^p<0.001^*^ | |
| **Oxygen saturation (%)** |  | |  | | | |  | |  | |
| Min. – Max. | 93.0 – 97.0 | | 75.0 – 89.0 | | | | 68.0 – 89.0 | | <0.001^*^ | |
| Mean ± SD. | 94.80±1.41 | | 85.60±3.23 | | | | 84.66±4.47 | |  |  |
| **Sig. bet. grps.** | p_1_<0.001^*^,p_2_<0.001^*^,p_3_=0.019^*^ | | | | | | | |  | |
| **Respiratory rate** |  | |  | | | |  | |  | |
| Min. – Max. | 16.0 – 31.0 | | 26.0 – 38.0 | | | | 27.0 – 36.0 | | <0.001^*^ | |
| Mean ± SD. | 22.55 ± 4.31 | | 31.16 ± 3.15 | | | | 32.0 ± 2.69 | |  |  |
| **Sig. bet. grps.** | p_1_<0.001^*^,p_2_<0.001^*^,p_3_=0.030^*^ | | | | | | | |  | |
| **CRP (mg/dL)** |  | |  | | | |  | |  | |
| Min. – Max. | 4.0 – 96.0 | | 4.0 – 114.0 | | | | 4.0 – 302.0 | | <0.001^*^ | |
| Median (IQR) | 24.0 (24.0 – 48.0) | | 48.0 (44.0 – 78.0) | | | | 48.0 (24.0 – 97.6) | |  |  |
| **Sig. bet. grps.** | p_1_<0.001^*^,p_2_<0.001^*^,p_3_=0.125 | | | | | | | |  | |
| **Lymphocytes (10^3^cell/µL)** |  | |  | | | |  | |  | |
| Min. – Max. | 0.54 – 2.90 | | 0.33 – 2.70 | | | | 0.23 – 2.80 | | <0.001^*^ | |
| Median (IQR) | 0.95 (0.70 – 2.35) | | 0.75 (0.62 – 0.87) | | | | 0.77 (0.65 – 0.99) | |  |  |
| **Sig. bet. grps.** | p_1_<0.001^*^,p_2_<0.001^*^,p_3_=0.082 | | | | | | | |  | |
| **Ferritin (ng/mL)** |  | | |  | |  | | |  |  |
| Min. – Max. | 116.0 – 1312.0 | | | 261.0 – 2618.80 | | 387.0 – 3600.0 | | | <0.001^*^ |  |
| Median (IQR) | 240.0 (166.0 – 322.0) | | | 480.0 (385.0 – 866.0) | | 865.0 (566.0–1322.0) | | |  |  |
| **Sig. bet. grps.** | p_1_<0.001^*^,p_2_<0.001^*^,p_3_<0.001^*^ | | | | | | | |  |  |
| **D dimer (ng/mL)** |  | | |  | |  | | |  |  |
| Min. – Max. | 0.42 – 960.0 | | | 0.28 – 2800.0 | | 0.80 – 7350.0 | | | <0.001^*^ |  |
| Median (IQR) | 10.70 (0.80 – 55.0) | | | 344.0 (7.84 – 874.0) | | 761.0 (312.0–2830.0) | | |  |  |
| **Sig. bet. grps.** | p_1_<0.001^*^,p_2_<0.001^*^,p_3_<0.001^*^ | | | | | | | |  |  |
| **Radiological** |  | | |  | |  | | |  |  |
| **CT** |  | | |  | |  | | |  |  |
| CO-RADS2 | 12 (10.0%) | | | 0 (0.0%) | | 0 (0.0%) | | | ^MC^p <0.001^*^ |  |
| CO-RADS3 | 72 (60.0%) | | | 27 (12.9%) | | 0 (0.0%) | | |  |  |
| CO-RADS4 | 36 (30.0%) | | | 98 (46.7%) | | 76 (40.0%) | | |  |  |
| CO-RADS5 | 0 (0.0%) | | | 85 (40.5%) | | 114 (60.0%) | | |  |  |

IQR: **Inter quartile range** SD: **Standard deviation F**: **F for One way ANOVA test**, Pairwise comparison bet. each 2 groups was done using **Post Hoc Test (Tukey)**

χ^2^: **Chi square test** MC: **Monte Carlo**

H: H for **Kruskal Wallis test,** Pairwise comparison bet. each 2 groups was done using **Post Hoc Test (Dunn's for multiple comparisons test)**

p: p value for comparing between the three studied groups

p_1_: p value for comparing between **Mild/moderate** and **Severe**

p_2_: p value for comparing between **Mild/moderate** and **Critical**

p_3_: p value for comparing between **Severe** and **Critical**

*: Statistically significant at p ≤ 0.05

**^#^**: WHO, 2020 classification

**ARDS**: acute respiratory distress syndrome, **AKI**: acute kidney injury**, CRP**: C reactive protein

**Supplementary Table 3:** **Correlation between 1^st^ reading IL-6 levels and laboratory parameters in selected patients (n = 160)**

| **Parameters** | **1^st^ reading IL-6 levels (pg\ml)** | |
| --- | --- | --- |
|  | **Spearman coefficient (r_s)_** | **P value** |
| **Age (years)** | 0.268^*^ | 0.001^*^ |
| **Hb** | -0.068 | 0.392 |
| **WBCS** | 0.261^*^ | 0.001^*^ |
| **Platelets** | 0.148 | 0.062 |
| **Lymphocytes** | -0.175 | 0.027^*^ |
| **CRP** | 0.199 | 0.012^*^ |
| **Ferritin** | 0.329^*^ | <0.001^*^ |
| **D dimer** | 0.186^*^ | 0.018^*^ |
| **LDH** | 0.312^*^ | <0.001^*^ |
| **ALT** | -0.003 | 0.974 |
| **AST** | 0.133 | 0.094 |
| **BUN** | 0.118 | 0.138 |
| **Creatinine** | 0.004 | 0.957 |
| **CO-RADS** | 0.186^*^ | 0.018^*^ |
| **Respiratory rate** | 0.405 | <0.001^*^ |
| **Oxygen saturation** | -0.464 | <0.001^*^ |

**r_s_: Spearman coefficient**

*: Statistically significant at p ≤ 0.05

**Supplementary Table 4:** **Relation between** **1^st^ reading IL6 levels and rs2285666 \ rs12252 genotypes and alleles in studied patients (n= 160)**

|  |  | **N** | **(pg\ml)** | | **Test of Sig.** | **P** |
| --- | --- | --- | --- | --- | --- | --- |
|  |  |  | **Mean ± SD.** | **Median  (Min. – Max.)** |  |  |
| **rs2285666** | **Genotype** |  |  |  |  |  |
|  | C\C | **50** | 62.75 ±84.72 | 18.0 (2.45 –351.0) | H= 6.827^*^ | 0.033^*^ |
|  | C\T | **42** | 111.5 ±127.3 | 70.0 (3.0 –484.0) |  |  |
|  | T\T | **68** | 89.06 ±113.4 | 32.95 (4.80 –531.0) |  |  |
|  | **Pairwise** |  | p_1_=0.010^*^, p_2_=0.081, p_3_=0.279 | |  |  |
|  | **Allele** |  |  |  |  |  |
|  | C | **142** | 77.16 ±101.0 | 31.0 (2.45 –484.0) | U= 11058.0 | 0.055 |
|  | T | **178** | 94.34 ± 116.6 | 39.0 (3.0 –531.0) |  |  |
| **rs12252** | **Genotype** |  |  |  |  |  |
|  | A\A | **51** | 66.18 ±81.90 | 47.0 (2.45 –351.0) | H= 2.744 | 0.254 |
|  | A\G | **46** | 99.38 ±127.5 | 39.0 (3.40 –531.0) |  |  |
|  | G\G | **63** | 94.09 ±116.0 | 31.20 (3.0 –484.0) |  |  |
|  | **Allele** |  |  |  |  |  |
|  | A | **148** | 76.50 ± 98.88 | 43.0 (2.45 –531.0) | U= 11388.0 | 0.104 |
|  | G | **172** | 95.51 ±118.5 | 34.70 (3.0 –531.0) |  |  |

SD: **Standard deviation U: Mann Whitney test**

H: H for **Kruskal Wallis test,** Pairwise comparison bet. each 2 groups were done using **Post Hoc Test (Dunn's for multiple comparisons test)**

p: p value for relation between **1^st^ reading IL6 level** and **genotypes**

p_1_: p value for relation between **C\C** and **C/T**

p_2_: p value for relation between **C\C** and **T/T**

p_3_: p value for relation between **C\T** and **T/T**

*: Statistically significant at p ≤ 0.05

**Supplementary Table 5:** **Relation between COVID-19 severity and** **the most prominent post COVID syndrome presentations** **among the two studied waves**

| **Post COVID  presentations** | **Mild/moderate** | | **Severe** | | **Critical** | | **P** |
| --- | --- | --- | --- | --- | --- | --- | --- |
|  | **No.** | **%** | **No.** | **%** | **No.** | **%** |  |
| **Group I: patients in wave 3**  **(n = 380)** | **(n = 80)** | | **(n = 150)** | | **(n = 150)** | |  |
| Cough | 32 | 40.0 | 20 | 13.3 | 0 | 0.0 | <0.001^*^ |
| Dyspnea | 24 | 30.0 | 0 | 0.0 | 0 | 0.0 | <0.001^*^ |
| Headache | 16 | 20.0 | 0 | 0.0 | 0 | 0.0 | <0.001^*^ |
| Fatigue, myalgia | 16 | 20.0 | 10 | 6.7 | 0 | 0.0 | <0.001^*^ |
| Bon ache | 8 | 10.0 | 0 | 0.0 | 0 | 0.0 | ^MC^p <0.001^*^ |
| Invasive fungal infection (Mucor mycosis) | 0 | 0.0 | 20 | 13.3 | 20 | 13.3 | 0.003^*^ |
| **Group II: patients in wave 4 (n = 520)** | **(n = 120)** | | **(n = 210)** | | **(n = 190)** | |  |
| Cough | 12 | 10.0 | 14 | 6.7 | 0 | 0.0 | <0.001^*^ |
| Dyspnea | 48 | 40.0 | 14 | 6.7 | 0 | 0.0 | <0.001^*^ |
| Headache | 12 | 10.0 | 14 | 6.7 | 0 | 0.0 | <0.001^*^ |
| Fatigue, and myalgia | 36 | 30.0 | 15 | 7.1 | 0 | 0.0 | <0.001^*^ |
| Bon ache | 12 | 10.0 | 15 | 7.1 | 0 | 0.0 | <0.001^*^ |
| Invasive fungal infection (Mucor mycosis) | 12 | 10.0 | 0 | 0.0 | 25 | 13.2 | <0.001^*^ |

χ^2^: **Chi square test** MC: **Monte Carlo**

p: p value for relation between the severity and outcome

*: Statistically significant at p ≤ 0.05

**Supplementary Table 6** Relation between ACE-2 (rs2285666) genotypes and clinical data in studied patients (n = 160)**.**

| **Parameters** | **rs2285666 genotype** | | | | | | **P** |
| --- | --- | --- | --- | --- | --- | --- | --- |
|  | **CC (n = 50)** | | **CT (n = 42)** | | **TT (n = 68)** | |  |
|  | **No.** | **%** | **No.** | **%** | **No.** | **%** |  |
| **Clinical presentation** |  |  |  |  |  |  |  |
| Fever | 32 | 64.0 | 30 | 71.4 | 48 | 70.6 | 0.680 |
| Cough | 28 | 56.0 | 24 | 57.1 | 42 | 61.8 | 0.796 |
| Dyspnea | 20 | 40.0 | 28 | 66.7 | 48 | 70.6 | 0.002^*^ |
| Headache | 10 | 20.0 | 4 | 9.5 | 8 | 11.8 | 0.286 |
| Chest pain | 6 | 12.0 | 2 | 4.8 | 8 | 11.8 | 0.419 |
| Vomiting or nausea | 10 | 20.0 | 2 | 4.8 | 12 | 17.6 | 0.090 |
| Diarrhea | 8 | 16.0 | 4 | 9.5 | 12 | 17.6 | 0.496 |
| Myalgia or arthralgia | 16 | 32.0 | 12 | 28.6 | 10 | 14.7 | 0.064 |
| Disturbed conscious level | 4 | 8.0 | 8 | 19.0 | 14 | 20.6 | 0.159 |
| **Complications** |  |  |  |  |  |  |  |
| ARDS | 10 | 20.0 | 18 | 42.9 | 40 | 58.8 | <0.001^*^ |
| AKI | 10 | 20.0 | 12 | 28.6 | 22 | 32.4 | 0.327 |
| Sepsis | 2 | 4.0 | 2 | 4.8 | 16 | 23.5 | 0.001^*^ |
| **Outcome** |  |  |  |  |  |  |  |
| Recovery, discharge | 38 | 76.0 | 14 | 33.3 | 20 | 29.4 | <0.001^*^ |
| ICU admission | 14 | 28.0 | 28 | 66.7 | 46 | 67.6 | <0.001^*^ |
| Death | 11 | 22.0 | 20 | 47.6 | 46 | 67.6 | <0.001^*^ |
| Post COVID symptoms | 33 | 66.0 | 8 | 19.0 | 9 | 13.2 | <0.001^*^ |

**Abbreviations:** CC: wild CT: hetero TT: mutant

*: Statistically significant at p ≤ 0.05

**Supplementary Table 7** Relation between IFITM3 (rs12252) genotypes and clinical data in studied patients (n = 160).

| **Parameters** | **rs12252 genotype** | | | | | | **P** |
| --- | --- | --- | --- | --- | --- | --- | --- |
|  | **AA (n = 51)** | | **AG (n = 46)** | | **GG (n = 63)** | |  |
|  | **No.** | **%** | **No.** | **%** | **No.** | **%** |  |
| **Clinical presentation** |  |  |  |  |  |  |  |
| Fever | 31 | 60.8 | 35 | 76.1 | 44 | 69.8 | 0.260 |
| Cough | 29 | 56.9 | 30 | 65.2 | 35 | 55.6 | 0.567 |
| Dyspnea | 21 | 41.2 | 28 | 60.9 | 47 | 74.6 | 0.001^*^ |
| Headache | 11 | 21.6 | 5 | 10.9 | 6 | 9.5 | 0.142 |
| Chest pain | 8 | 15.7 | 2 | 4.3 | 6 | 9.5 | 0.175 |
| Vomiting or nausea | 9 | 17.6 | 4 | 8.7 | 11 | 17.5 | 0.365 |
| Diarrhea | 7 | 13.7 | 6 | 13.0 | 11 | 17.5 | 0.778 |
| Myalgia or arthralgia | 16 | 31.4 | 7 | 15.2 | 15 | 23.8 | 0.175 |
| Disturbed conscious level | 8 | 15.7 | 8 | 17.4 | 10 | 15.9 | 0.969 |
| **Complications** |  |  |  |  |  |  |  |
| ARDS | 13 | 25.5 | 22 | 47.8 | 33 | 52.4 | 0.011^*^ |
| AKI | 11 | 21.6 | 10 | 21.7 | 23 | 36.5 | 0.121 |
| Sepsis | 8 | 15.7 | 4 | 8.7 | 8 | 12.7 | 0.581 |
| **Outcome** |  |  |  |  |  |  |  |
| Recovery, discharge | 40 | 78.4 | 15 | 32.6 | 17 | 27.0 | <0.001^*^ |
| ICU admission | 15 | 29.4 | 30 | 65.2 | 43 | 68.3 | <0.001^*^ |
| Death | 10 | 19.6 | 26 | 56.5 | 41 | 65.1 | <0.001^*^ |
| Post COVID symptoms | 34 | 66.7 | 9 | 19.6 | 7 | 11.1 | <0.001^*^ |

**Abbreviations:** AA: wild AG: hetero GG: mutant

*: Statistically significant at p ≤ 0.05

**Supplementary Table 8 Comparison between the three studied groups and control according to rs2285666 in different genetic models**

| **SNPs** | **Mild/ moderate  (n = 40)** | | **Severe  (n = 60)** | | **Critical (n = 60)** | | **Control (n = 40)** | | **P** |
| --- | --- | --- | --- | --- | --- | --- | --- | --- | --- |
|  | **No.** | **%** | **No.** | **%** | **No.** | **%** | **No.** | **%** |  |
| **Dominant (CT+TT vs. CC)** | 10/40 | 25.0 | 48/60 | 80.0 | 52/60 | 86.7 | 10/40 | 25.0 | <0.001^*^ |
| **p_0_** | 1.000 | | <0.001^*^ | | <0.001^*^ | |  |  |  |
| **Sig. bet. grps.** | p_1_<0.001^*^,p_2_<0.001^*^,p_3_=0.327 | | | | | |  |  |  |
| **Recessive (TT vs. CC+CT)** | 4/40 | 10.0 | 32/60 | 53.3 | 32/60 | 53.3 | 2/40 | 5.0 | <0.001^*^ |
| **p_0_** | ^FE^p=0.675 | | <0.001^*^ | | <0.001^*^ | |  |  |  |
| **Sig. bet. grps.** | p_1_<0.001^*^,p_2_<0.001^*^,p_3_=1.000 | | | | | |  |  |  |
| **Co-dominant-1 (CT vs. CC)** | 6/36 | 16.7 | 16/28 | 57.1 | 20/28 | 71.4 | 8/38 | 21.1 | <0.001^*^ |
| **p_0_** | 0.630 | | 0.003^*^ | | <0.001^*^ | |  |  |  |
| **Sig. bet. grps.** | p_1_=0.001^*^,p_2_<0.001^*^,p_3_=0.265 | | | | | |  |  |  |
| **Co-dominant-2 (TT vs. CC)** | 4/34 | 11.8 | 32/44 | 72.7 | 32/40 | 80.0 | 2/32 | 6.3 | <0.001^*^ |
| **p_0_** | ^FE^p=0.673 | | <0.001^*^ | | <0.001^*^ | |  |  |  |
| **Sig. bet. grps.** | p_1_<0.001^*^,p_2_<0.001^*^,p_3_=0.434 | | | | | |  |  |  |
| **Over dominant (CT vs. CC+TT)** | 6/40 | 15.0 | 16/60 | 26.7 | 20/60 | 33.3 | 8/40 | 20.0 | 0.173 |
| **p_0_** | 0.556 | | 0.444 | | 0.146 | |  |  |  |
| **Sig. bet. grps.** | p_1_=0.168,p_2_=0.041^*^,p_3_=0.426 | | | | | |  |  |  |

χ^2^: **Chi square test** FE**: Fisher Exact**  MC: **Monte Carlo**

p_0_: p value for **Chi square test** for comparing between **Control** and each other groups

p_1_: p value for **Chi square test** for comparing between **Mild/moderate** and **Severe**

p_2_: p value for **Chi square test** for comparing between **Mild/moderate** and **Critical**

p_3_: p value for **Chi square test** for comparing between **Severe** and **Critical**

*: Statistically significant at p ≤ 0.05

**Supplementary Table 9 Comparison between the three studied groups and control according to rs12252 in different genetic models**

|  | **Mild/ moderate  (n = 40)** | | **Severe  (n = 60)** | | **Critical (n = 60)** | | **Control (n = 40)** | | **P** |
| --- | --- | --- | --- | --- | --- | --- | --- | --- | --- |
|  | **No.** | **%** | **No.** | **%** | **No.** | **%** | **No.** | **%** |  |
| **Dominant (AG+GG vs. AA)** | 12/40 | 30.0 | 48/60 | 80.0 | 49/60 | 81.7 | 10/40 | 25.0 | <0.001^*^ |
| **p_0_** | 0.617 | | <0.001^*^ | | <0.001^*^ | |  |  |  |
| **Sig. bet. grps.** | p_1_<0.001^*^,p_2_<0.001^*^,p_3_=0.817 | | | | | |  |  |  |
| **Recessive (GG vs. AA+AG)** | 2/40 | 5.0 | 36/60 | 60.0 | 25/60 | 41.7 | 2/40 | 5.0 | <0.001^*^ |
| **p_0_** | ^FE^p=1.0 | | <0.001^*^ | | <0.001^*^ | |  |  |  |
| **Sig. bet. grps.** | p_1_<0.001^*^,p_2_<0.001^*^,p_3_=0.045^*^ | | | | | |  |  |  |
| **Co-dominant-1 (AG vs. AA)** | 10/38 | 26.3 | 12/24 | 50.0 | 24/35 | 68.6 | 8/38 | 21.1 | <0.001^*^ |
| **p_0_** | 0.589 | | 0.018^*^ | | <0.001^*^ | |  |  |  |
| **Sig. bet. grps.** | p_1_=0.058,p_2_<0.001^*^,p_3_=0.151 | | | | | |  |  |  |
| **Co-dominant-2 (GG vs. AA)** | 2/30 | 6.7 | 36/48 | 75.0 | 25/36 | 69.4 | 2/32 | 6.3 | <0.001^*^ |
| **p_0_** | ^FE^p=1.0 | | <0.001^*^ | | <0.001^*^ | |  |  |  |
| **Sig. bet. grps.** | p_1_<0.001^*^,p_2_<0.001^*^,p_3_=0.572 | | | | | |  |  |  |
| **Over dominant (AG vs. AA+GG)** | 10/40 | 25.0 | 12/60 | 20.0 | 24/60 | 40.0 | 8/40 | 20.0 | 0.052 |
| **p_0_** | 0.592 | | 1.000 | | 0.036^*^ | |  |  |  |
| **Sig. bet. grps.** | p_1_=0.554,p_2_=0.121,p_3_=0.017^*^ | | | | | |  |  |  |

χ^2^:**Chi square test** MC: **Monte Carlo**

p_0_: p value for **Chi square test** for comparing between **Control** and each other groups

p_1_: p value for **Chi square test** for comparing between **Mild/moderate** and **Severe**

p_2_: p value for **Chi square test** for comparing between **Mild/moderate** and **Critical**

p_3_: p value for **Chi square test** for comparing between **Severe** and **Critical**

*: Statistically significant at p ≤ 0.05
